# Inhibition of K^+^ Transport through Na^+^, K^+^-ATPase by Capsazepine: Role of Membrane Span 10 of the α-Subunit in the Modulation of Ion Gating

**DOI:** 10.1371/journal.pone.0096909

**Published:** 2014-05-09

**Authors:** Yasser A. Mahmmoud, Michael Shattock, Flemming Cornelius, Davor Pavlovic

**Affiliations:** 1 Department of Biomedicine, University of Aarhus, DK-8000 Aarhus C, Denmark; 2 Cardiovascular Division, King's College London, The Rayne Institute, St. Thomas' Hospital, London, United Kingdom; University of Geneva, Switzerland

## Abstract

Capsazepine (CPZ) inhibits Na^+^,K^+^-ATPase-mediated K^+^-dependent ATP hydrolysis with no effect on Na^+^-ATPase activity. In this study we have investigated the functional effects of CPZ on Na^+^,K^+^-ATPase in intact cells. We have also used well established biochemical and biophysical techniques to understand how CPZ modifies the catalytic subunit of Na^+^,K^+^-ATPase. In isolated rat cardiomyocytes, CPZ abolished Na^+^,K^+^-ATPase current in the presence of extracellular K^+^. In contrast, CPZ stimulated pump current in the absence of extracellular K^+^. Similar conclusions were attained using HEK293 cells loaded with the Na^+^ sensitive dye Asante NaTRIUM green. Proteolytic cleavage of pig kidney Na^+^,K^+^-ATPase indicated that CPZ stabilizes ion interaction with the K^+^ sites. The distal part of membrane span 10 (M10) of the α-subunit was exposed to trypsin cleavage in the presence of guanidinum ions, which function as Na^+^ congener at the Na^+^ specific site. This effect of guanidinium was amplified by treatment with CPZ. Fluorescence of the membrane potential sensitive dye, oxonol VI, was measured following addition of substrates to reconstituted inside-out Na^+^,K^+^-ATPase. CPZ increased oxonol VI fluorescence in the absence of K^+^, reflecting increased Na^+^ efflux through the pump. Surprisingly, CPZ induced an ATP-independent increase in fluorescence in the presence of high extravesicular K^+^, likely indicating opening of an intracellular pathway selective for K^+^. As revealed by the recent crystal structure of the E_1_.AlF_4_^-^.ADP.3Na^+^ form of the pig kidney Na^+^,K^+^-ATPase, movements of M5 of the α-subunit, which regulate ion selectivity, are controlled by the C-terminal tail that extends from M10. We propose that movements of M10 and its cytoplasmic extension is affected by CPZ, thereby regulating ion selectivity and transport through the K^+^ sites in Na^+^,K^+^-ATPase.

## Introduction

The Na^+^,K^+^-ATPase (sodium pump) is a heterodimeric membrane protein that pumps three sodium ions out- and two potassium ions into the cell, at the expenditure of the energy derived from hydrolysis of one ATP molecule. Consequently Na^+^,K^+^-ATPase establishes an electrochemical gradient for these ions across the plasma membrane, which is indispensable for many cell functions [1]. The enzyme consists of a catalytic α-subunit that undergoes ion- and ATP-dependent conformational transitions coupling ATP hydrolysis to the up-hill transport of ions. The α-subunit is associated with a glycosylated β-subunit that is important for function, folding, and plasma membrane delivery of the enzyme complex [2]. The α-subunit contains a highly conserved site for inhibition by cardiac glycosides [3]. Small auxiliary proteins belonging to the FXYD family interact with and regulate Na^+^,K^+^-ATPase activity in several tissues [4]. The Na^+^,K^+^-ATPase belongs to P_2_-type ATPases, which includes three closely related members; sarcoplasmic reticulum Ca^2+^-ATPase (SERCA), gastric and colonic H^+^, K^+^-ATPases [5].

Crystal structures of Na^+^,K^+^-ATPase in the two major conformations [6]–[9] have provided significant structure-function information. In addition, electrophysiological [10], [11] and biophysical [12] measurements have proven to be pivotal to understanding the events associated with ion binding and release, as well as voltage-dependence of the pump. The mechanism of Na^+^,K^+^-ATPase is described by the Post-Albers scheme, which involves binding of three Na^+^ ions from the cytoplasmic side (E_1_ form), providing the trigger for phosphoryl transfer and formation of E_1_P(3Na^+^). The three Na^+^ ions are generally considered to be first released to the extracellular side in a sequential manner from E_2_P(3Na^+^) [10] and then 2K^+^ bind at the extracellular side, where K^+^ occlusion stimulates phosphoenzyme hydrolysis, and hence E_2_(2K^+^) is formed. K^+^ release to the cytoplasm and shift to the E_1_ form is facilitated by ATP. In contrast to SERCA1a [5], crystal structures of the Na^+^,K^+^-ATPase revealed that the C-terminal tail of the α-subunit has a unique position, interacting with the ion binding core [7], [9]. The recent structure of the E_1_.AlF_4_^-^.ADP.3Na^+^ form indicates that the C-terminal tail functions as a controller of the movement of M5 [9]. Mutational studies have shown that the C-terminal tail plays an important role in the regulation of Na^+^ interaction from both sides of the membrane [13]. In this regard, mutations in this domain have been shown to stabilize ion occlusion at the shared sites [14] and also to modify ion interaction with the Na^+^ unique site [15].

We have previously documented an unprecedented mode of sodium pump inhibition. Capsazepine (CPZ), a synthetic transient receptor potential vanilloid antagonist [16], inhibits K^+^-dependent ATP hydrolysis with no effect on Na^+^-ATPase (see below) activity [17]. CPZ treatment of Na^+^,K^+^-ATPase was found to strongly modify nucleotide interaction with the pump and stabilize K^+^ occlusion without affecting ^22^Na^+^ influx through inside-out pumps reconstituted in lipid vesicles [17]. Hence it was concluded that CPZ blocks the K^+^ transport half-cycle through the pump, leaving a Na^+^ half-cycle intact. Such deviation from normal transport mode is likely a consequence of modified ion binding, raising the interesting possibility that the Na^+^ specific site may function independently of the shared sites; thereby pump stoichiometry may be modulated. In support of this idea, early studies have reported a decrease in the coupling ratio of the pump in the presence of low cytoplasmic Na^+^ concentrations (Ref. [18], and see Discussion). In addition, several spontaneous pump mutants were identified in disease; kinetic analyses of some of these mutants revealed a severely modified binding of Na^+^ with no effect on the K^+^ interaction [19]. Sodium-dependent activity is a partial reaction of the Na^+^,K^+^-ATPase that occurs in the absence of external K^+^ [20], referred to as Na^+^-ATPase activity, where Na^+^, Na^+^-exchange is believed to occur at 3Na^+^:2 Na^+^ stoichiometry. Na^+^ replaces extracellular K^+^ but with low affinity, and hence the maximum velocity of Na^+^-ATPase is typically 5–15% of Na^+^,K^+^-ATPase in most preparations.

In this study, we present novel information on the modification of Na^+^,K^+^-ATPase by CPZ. First, the inhibition of pump mediated K^+^ transport and stimulation of Na^+^ transport (in the absence of extracellular K^+^) was tested and confirmed using different experimental setups; namely, by measuring 1) whole cell sodium pump current in isolated cardiomyocytes, and 2) dynamic changes in intracellular Na^+^ concentration using the dye Asante NaTRIUM green II (ASG II). We have also performed hydrolytic activity measurements and proteolytic cleavage experiments to investigate ion-dependent conformational changes. Finally, oxonol VI, a membrane potential probe, was used to investigate the effect of CPZ on pump mediated charge movements. These different experimental approaches were all employed to shed light on the possible mechanism of action of this drug. Our data, in combination with prior mutagenesis studies, strongly suggest an uncoupled mode of the Na^+^,K^+^-ATPase in which movement of M10, which carries the C-terminal tail, blocks K^+^ transport but allows Na^+^ efflux.

## Materials and Methods

### Cell isolation and culture of adult rat ventricular myocytes

Animals were maintained humanely in compliance with the “Principles of Laboratory Animal Care” formulated by the National Society for Medical Research and the Guide for Care and Use of Laboratory Animals prepared by the National Academy of Sciences and published by the National Institutes of Health (NIH Pub. No. 85–23, revised 1985). All animal protocols were approved both by the local King's College Ethical Review Process Committee and by the UK Government Home Office (Animals Scientific Procedures Group). Animals were anesthetized with sodium pentobarbital in combination with sodium heparin (200 mg· kg^−1^ and 200 IU· kg^−1^, respectively). Ventricular myocytes (ARVM) were isolated from the hearts of adult male Wistar rats (200–250 g, B&K Universal) by standard collagenase enzymatic digestion as described earlier [21]. In all of the electrophysiology experiments, myocytes were used two hours post-isolation.

### Electrophysiology

Adult rat ventricular myocytes were voltage-clamped and Na^+^/K^+^ pump current (I_p_) was recorded at 35°C using the whole-cell ruptured-patch technique as described previously [21]–[23]. Briefly, myocytes were studied under whole-cell voltage-clamp using electrodes which had resistances of 1–2 MΩ when filled with (in mM) 95 CsCH_3_O_3_S, 25 NaCH_3_O_3_, 20 CsCl, 1 MgCl_2_, 1.5 CaCl_2_, 10 HEPES, 5 EGTA, 5 MgATP, 5 creatine phosphate pH 7.2. External solution was (in mM) 140 NaCl, 1 MgCl_2_, 2 NiCl_2_, 1 BaCl_2_, 5 KCl, 10 Glucose, 10 HEPES, pH 7.2 at 35°C. The solution with either dimethyl sulfoxide (DMSO) vehicle or 30 µM CPZ was flowing at a rate of 3 ml·min^-1^. Cell capacitance was measured from the capacitance transient generated on application of a voltage step from the holding potential of −90 mV to −80 mV. Current generated was recorded via an Axopatch 200A amplifier and pClamp10 software (Molecular Devices, California, U.S.A). Passive K^+^ currents are routinely blocked by replacing intracellular K^+^ with pipette Cs and by the addition of 1 mM Ba^2+^ to the extracellular solution.

### Intracellular Na^+^ fluorescence measurements

For measurement of changes in intracellular Na^+^ a similar protocol was employed as described previously [24]. HEK293 cells were plated in black 96 well ViewPlate (PerkinElmer Inc., USA) and cultured in Dulbecco's Modified Eagle Medium (DMEM) in the presence of 10% fetal calf serum and antibiotics (100 IU ml^−1^ of penicillin and 100 µg·ml^−1^ streptomycin) until confluent. DMEM was replaced with Tyrode (in mmol/L: 140 NaCl, 4 KCl, 1 MgCl_2_, 1 CaCl_2_, 10 HEPES, and 10 glucose, pH 7.4) and HEK293 cells loaded for 2 hours with 5 µM of the sodium fluorophore ASG II (Teflabs Inc., USA), in the presence of the non-ionic surfactant Pluronic F-127 (0.05% w/v). External fluorophore was then washed out and cytosolic fluorophore was allowed to de-esterify for 20 min before proceeding with intracellular Na^+^ measurements. HEK293 cells were de-esterified in Tyrode solution and the effects of CPZ on intracellular Na^+^ examined in either K^+^-free (in mmol/L: 140 NaCl, 2 EGTA, 10 HEPES, and 10 glucose, pH 7.4) or K^+^-containing solution (in mmol/L: 140 NaCl, 4 KCl, 2 EGTA, 10 HEPES, and 10 glucose, pH 7.4). Single excitation (485 nm) single emission (535 nm) (dichroic 515 nm) measurements were performed using a Gemini XPS Fluorescence Microplate Reader (Molecular Devices, USA), at room temperature. Fluorescence signal decline represents overall Na^+^ efflux, whereas, an increase in fluorescence signal represents Na^+^ influx [24], [25].

### Na^+^,K^+^-ATPase preparation and hydrolytic activity

Plasma membranes from pig kidney red outer medulla were isolated according to a modified Jørgensen's Materials and Methods described earlier [26]. In brief, minced tissue pieces were homogenized in 30 mM imidazole buffer pH 7.4, containing 250 mM sucrose and 1 mM EDTA. The homogenate was subjected to several differential centrifugation steps to obtain a microsomal plasma membrane fraction. Microsomes were treated with a mild concentration of sodium dodecyl sulphate (SDS) to open sealed vesicles and dissociate several peripheral proteins from the membrane. The final preparation had a specific activity of 1.8–2.2 mmol.h^−1^.mg^−1^ protein at 37°C. Protein concentration was determined using a BioRad detergent compatible kit (Cat# 500–0113), according to the manufacturer's instructions. ATPase activity was measured by incubating the enzyme with buffer and substrates (see separate figure legends) at 24°C followed by measuring phosphate liberated from ATP, according to the method of Baginsky [27]. Control activities were measured in identical conditions but in the presence of 1 mM ouabain. For the temperature effect and vanadate sensitivity measurements, a sigmoid dose-response function was used:
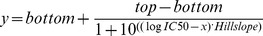
IC50 is the agonist concentration that gives a response halfway between top and bottom.

*Para*-nitrophenyl phosphatase (*p*NPPase) activity was measured in a reaction mixture containing 30 mM histidine buffer, 5 mM MgCl, 10 mM *p*NPP (disodium salt), 5 µg protein, and 100 mM of either NaCl or KCl. The pH was adjusted using Tris-HCl. The reactions were stopped with 4.5% trichloroacetic acid (TCA). Ouabain sensitive *para*-nitrophenol release was determined using a colorimetric method, by measuring the absorbance of the post-hydrolytic mixture at 410 nm. *para*-nitrophenol has a molar extinction coefficient of 1.8 · 10^4^ M^−1^ · cm^−1^.

### Proteolytic cleavage, gel electrophoresis, and immunoblotting

Controlled proteolysis of the pig kidney α-subunit was performed in a reaction mixture containing 100 µg of purified membrane protein suspended in 25 mM histidine pH 7.0 in the presence of different additions as described in figure legends. The protein was preincubated for 30 min at 24 °C before the addition of 2 µg of proteinase K (PK), and the mixtures were incubated for 45 min. Proteolysis was terminated with an equal volume of SDS sample buffer containing 1% TCA to irreversibly inhibit the protease. Cleavage with trypsin was performed in the same way but buffers with different pH values were used. The mixtures were analyzed by SDS-PAGE overnight at 150 V and 12 mA per gel. 10 µg of protein was loaded onto 8% SDS-PAGE, and protein fragments on the gel were transferred to polyvinylidene fluoride (PVDF) membranes and visualized by Western blotting using a C-terminal α-subunit antibody [raised against peptide I1002-Y1016 of the pig kidney α-subunit], as described previously [28]. Trypsin [29] and PK [30] cleavage patterns of the Na^+^,K^+^-ATPase α-subunit were previously characterized. We have produced new affinity-purified antibody raised against the last 5 C-terminal residues of the α-subunit (KETYY).

### Reconstitution of Na^+^,K^+^-ATPase

Reconstitution of Na^+^,K^+^-ATPase was performed as described previously [31]. Phosphatidyl choline/cholesterol vesicles containing shark Na^+^,K^+^-ATPase were prepared in 30 mM histidine buffer pH 7, 2 mM MgCl_2_, and either 30 mM NaCl alone or together with 30 mM KCl. Following treatment with C_12_E_8_ (detergent:protein ratio of 2∶1) and isolation of the solubilized protein by centrifugation, the soluble protein was mixed with phosphatidyl choline/cholesterol in C_12_E_8_ and liposomes were formed by overnight incubation with biobeads to remove the detergent.

### Fluorescence measurements

A SPEX Fluorolog-3 spectrofluorometer equipped with a thermostated cell compartment (23°C) and a magnetic stirrer was used to measure changes in membrane potential across liposomal membrane [30], [32]. 50 µl proteoliposomes were added to 3 ml cuvette containing 30 mM histidine buffer pH 7.2, 2 mM MgCl_2_, 75 µM ATP, 0.5 µM oxonol VI, as well as other additions (see figure legends). The ATP powered increase in fluorescence emission at 660 nm (slit width 5 nm), indicative of ion translocation across the membrane, is detected following excitation of the dye at 580 nm (slit width 20 nm). Data were analyzed using a one-phase association function:
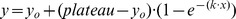

*k* denotes the rate constant of the reaction.

### Statistical analysis

Quantitative electrophysiology data are shown as mean ± standard error of the mean (SEM) of measurements derived from cells isolated from at least 6 individual hearts. Differences between experimental groups were tested by one-way ANOVA followed by a Bonferroni post-hoc test or by unpaired *t*-tests. Differences were considered significant at p<0.05. Proteolytic cleavage experiments were performed four times, with a representative result shown.

## Results

### Effects of CPZ on pump current in rat cardiomyocytes

**Figure 1:**
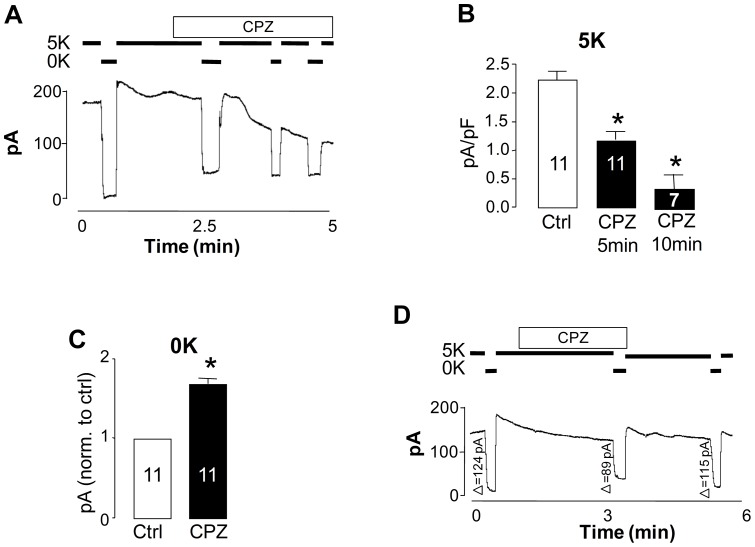
CPZ decreases K^+^-dependent Na^+^, K^+^-ATPase transport activity. **A.** Raw trace of the effects of 30 µM of CPZ on Na^+^, K^+^-ATPase I_p_ in adult rat cardiac myocytes. The horizontal bold lines indicate the addition and wash-off of 5 mM KCl or the addition of CPZ, as indicated. **B.** Steady state current obtained following 5 and 10 min incubation with CPZ, compared to controls not treated with CPZ. **C.** Changes in K^+^-independent I_p_ following the addition of CPZ. **D.** The CPZ-induced changes of Na^+^, K^+^-ATPase I_p_ in adult rat cardiac myocytes are reversible. The data represent cells isolated from at least 6 individual animals, (number of cells shown inside bars; 1 min time point not shown) and are expressed as mean ±SEM (*significantly different from control, *P*<0.05). Differences between experimental groups were tested by one-way ANOVA followed by a Bonferroni post-hoc test in panel B and by unpaired *t*-test in panel C.

In order to investigate the functional effects of CPZ on Na^+^,K^+^-ATPase activity in a more integrated system, pump current (I_p_) was measured in single freshly isolated adult rat ventricular cardiomyocytes using a whole-cell ruptured patch clamp technique [22], [23]. I_p_ was defined as current inhibited by removal of extracellular K^+^ (K^+^-dependent current). I_p_ was measured in Tyrode solution followed by treatment with either DMSO vehicle control or 30 µM CPZ. Perfusion of the cardiomyocytes with solution containing CPZ decreased K^+^-dependent current, as shown in Fig. 1A. Five min of perfusion with CPZ decreased I_p_ by 52% (I_p_ decreased from 2.246 ± 0.171 to 1.172±0.1607 pA/pF; p<0.05, n = 11), whereas, 10 min of perfusion with CPZ resulted in 85% inhibition (I_p_ = 0.3250±0.2136 pA/pF; p<0.05, n = 7), as shown in Fig. 1B.

In the absence of extracellular K^+^, the activity of the enzyme is confined to Na^+^ translocation steps in which extracellular Na^+^ acts like K^+^ by binding to the extracellular sites. In most systems, Na^+^, Na^+^ exchange occurs at a significantly lower rate (∼5–15%) compared to Na^+^, K^+^ exchange, owing to the difference in affinity between Na^+^ and K^+^ at the extracellular sites [20]. Interestingly, in the absence of extracellular K^+^, CPZ perfusion resulted in a small but significant increase in K^+^-independent I_p_ (Fig. 1C). This increase appears to occur on a faster timescale than the concomitant decrease in K^+^-dependent current (defined as K^+^-dependent I_p_ minus K^+^-independent I_p_). The effects of CPZ on I_p_ is reversible; washing off the CPZ with control solution partially restored K^+^-dependent I_p_ and reduced increase in K^+^-independent I_p_, as demonstrated by changes in I_p_ (shown to the left of each K^+^ to K^+^-free steps in Fig. 1D).

**Figure 2:**
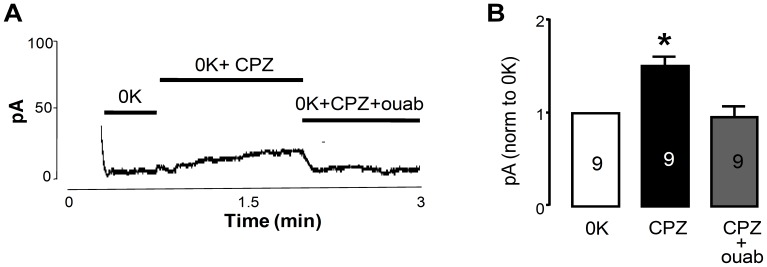
CPZ increases K^+^-independent Na^+^, K^+^-ATPase activity. **A**. Raw trace of the effects of treatment with 30 µM of CPZ on K^+^-independent I_p_ followed by the addition of 10 mM ouabain. **B**. Changes in steady state K^+^-independent I_p_ as a result of CPZ and CPZ plus ouabain, normalized to the K^+^-free control I_p_. The data represent cells isolated from 6 individual animals [number of cells are shown inside bars] and are expressed as mean ±SEM (*significantly difference from control, *P*<0.05). Differences between experimental groups were tested by one-way ANOVA followed by a Bonferroni post-hoc test.

In order to determine whether the increase in K^+^-independent I_p_ (Fig. 1C) is ouabain-sensitive, stimulation of K^+^-independent I_p_ by CPZ was allowed to occur and then ouabain (10 mM) was added. Ouabain completely inhibited the CPZ-induced increase in K^+^-independent I_p_ (Fig. 2).

### Effects of CPZ on intracellular Na^+^

**Figure 3:**
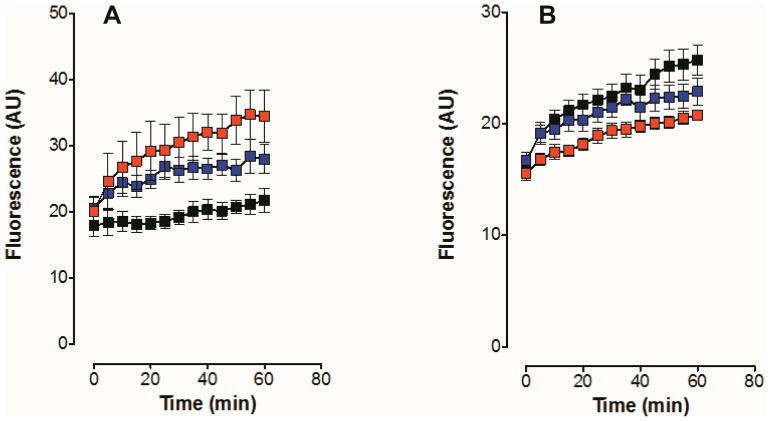
Effect of CPZ on ASG II fluorescence in cultured HEK293 cells. Cultured cells were suspended in Tyrode buffer and loaded with ASG II prior to fluorescence measurements, as described in Materials and Methods. Fluorescence was measured at 535 nm in the presence of 4 mM K^+^ (A), or in the absence of K^+^ (B) on the extracellular side. Extracellular Na^+^ was 140 mM in both cases. Fluorescence, indicative of changes in intracellular Na^+^ concentration, was measured as a function of time, following treatment of cells with DMSO (black squares), 10 µM CPZ (blue squares), or 30 µM CPZ (red squares).

The effects of CPZ on intracellular Na^+^ were examined in human embryonic kidney cells (HEK293) loaded with a Na^+^ sensitive dye, ASG II, using fluorescence spectroscopy [24], [25]. Fluorescence of ASG II increases linearly with intracellular Na^+^ over a physiological intracellular Na^+^ range 0–40 mM in primary astrocytes [33] and HEK293 cells (data not shown). In the presence of an extracellular K^+^ concentration of 4 mM (Fig. 3A), CPZ produced a dose-dependent increase in ASG II fluorescence compared to DMSO controls (∼170% following treatment with 30 µM CPZ over a 60 min period), consistent with increased intracellular Na^+^. This result is expected as CPZ was shown to inhibit coupled K^+^-dependent activity (Fig. 1) and hence the major route of Na^+^ extrusion from these cells is inhibited. In the absence of extracellular K^+^, the normal Na^+^/K^+^ translocation via the pump is inhibited and hence intracellular Na^+^ inevitably rises. In the absence of K^+^, however, CPZ reduced the rate of intracellular Na^+^ increase in a dose-dependent manner (Fig. 3B). This result is consistent with increased uncoupled Na^+^ efflux from cells as shown in Figs. 1C and 2.

### Effect of temperature, pH, and vanadate sensitivity of the CPZ modified enzyme

**Figure 4:**
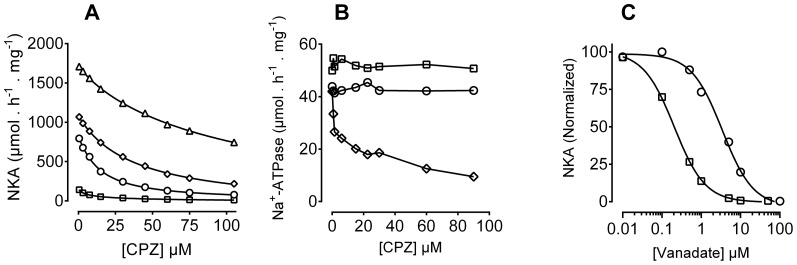
Effect of temperature, pH, and vanadate sensitivity of the CPZ treated enzyme. **A.** Pig kidney Na^+^,K^+^-ATPase activity was measured at four different temperatures, as described in Materials and Methods, in the presence of the indicated CPZ concentrations. The reaction contained 30 mM histidine buffer, pH 7.2, 100 mM NaCl, 20 mM KCl, 3 mM MgCl_2_, and 3 mM ATP. The inhibition curves were analyzed using the Hill equation, giving the following IC_50_ values; squares, 5°C (7.64±1.14 µM); circles, 15°C (13.88±1.05 µM); diamonds, 30°C (37.33±1.08 µM); and triangles, 37 °C (108.20±1.31 µM). **B.** Effect of pH on CPZ inhibition of Na^+^-ATPase activity. Na^+^-ATPase activity was measured as described in panel A, but in the absence of K^+^ and in the presence of different pH values (the reaction was buffered with 30 mM Tris adjusted with HCl), in the presence of the indicated CPZ concentrations. Squares, circles, and diamonds indicate measurements performed at pH 6, 7, or 8, respectively. **C**. Na^+^,K^+^-ATPase (NKA) activity was measured as in panel A, but in the presence of 1 mM ATP and the indicated vanadate concentrations at 23°C. The ATPase mixture contained either DMSO (squares) or 20 µM CPZ (circles).

CPZ inhibition curves were measured at different temperatures, showing that temperature strongly influences CPZ inhibition (Fig. 4A). The IC50 values for CPZ inhibition were 108.2±1.31 µM, 37.33±1.08 µM, 13.88±1.05 µM, and 7.64±1.14 µM at 37°C, 30°C, 15°C, and 5°C, respectively.

Previously, we have shown that Na^+^-ATPase activity is insensitive to CPZ [17]. We now demonstrate that Na^+^-ATPase activity is inhibited by CPZ at basic pH but not when the pH is neutral or acidic (Fig. 4B).

Vanadate sensitivity was studied to gain information on the conformational equilibrium of the pump [28]. Orthovanadate is a transition state analogue of inorganic phosphate that binds preferentially to the *E*_2_ conformation of P-type ATPases. Thus, the sensitivity of Na^+^,K^+^-ATPase to inhibition by vanadate reflects the proportion of the enzyme adopting an E_2_ conformation. Vanadate inhibition of the control enzyme followed a sigmoid curve. Analysis using the Hill equation indicated an IC_50_ for vanadate inhibition of 0.205±0.02 µM (Fig. 4C, squares). After treatment with CPZ a substantial decrease in vanadate sensitivity was observed (Fig. 4C, circles). The IC_50_ of inhibition by vanadate in the presence of CPZ was 3.43±1.05 µM. This 17 fold increase in IC_50_ indicates a CPZ-mediated strong stabilization of an E_1_-like conformation.

### CPZ inhibits K^+^-*p*NPPase but stimulates *p*NPP hydrolysis in the presence of Na^+^

**Figure 5:**
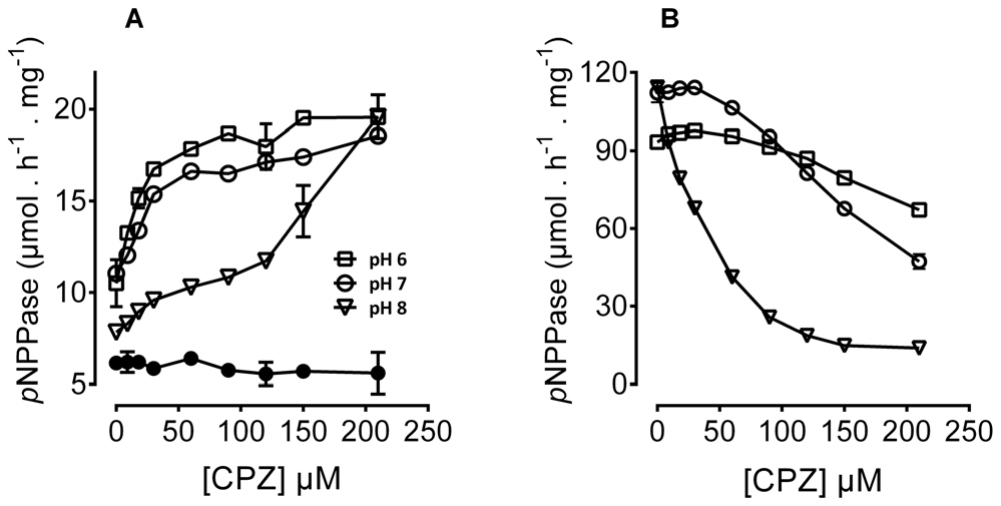
Effect of CPZ on *p*NPP hydrolysis by Na^+^,K^+^-ATPase. *p*NPPase activity was measured in the presence of histidine buffer (with the pH adjusted with Tris/HCl), 10 mM MgCl_2_, 10 mM Na_2_^+^-*p*NPP, 100 mM NaCl (A) or 100 mM KCl (B) in the presence of the indicated CPZ concentrations and in the absence of ATP. Released *p*NP was measured in a spectrophotometer at 410 nm as described under Materials and Methods. Data are expressed as µmol hydrolyzed *p*NPP · h^−1^ · mg^−1^ (at 24°C). The black circles depict the effect of linoleic acid on *p*NPPase activity in the presence of 100 mM Na^+^ at concentrations identical to those of CPZ. Note that the residual activity seen in panel B at high CPZ concentrations is likely due to the presence of 20 mM Na^+^ added with *p*NPP.

Na^+^,K^+^-ATPase hydrolyses *p*NPP in a partial reaction involving an E_2_ form, and is detected following incubation of the enzyme in the presence of K^+^ (E_2_K^+^ form), or in the presence of Na^+^ and MgATP (E_2_P form). *p*NPP hydrolysis is inhibited by ligands that stabilize the E_1_ conformation and is therefore not observed when Na^+^ substitutes K^+^. We have now found that CPZ stimulates *p*NPP hydrolysis in the presence of 100 mM Na^+^ and in the absence of K^+^ (Fig. 5A). The maximum level of stimulation was independent of pH although the apparent affinity of inhibition by CPZ decreased at basic pH. Linoleic acid, a free fatty acid that stabilizes the E_2_ conformation [32], does not produce a similar effect (Fig. 5A, closed circles). On the other hand, K^+^-dependent *p*NPPase is inhibited by CPZ at all pH values tested, but the inhibition was found to be strongly dependent on pH (Fig. 5B); the apparent affinity of inhibition by CPZ was much higher at basic pH compared to acidic and neutral pH.

### Cleavage with Proteinase K

**Figure 6:**
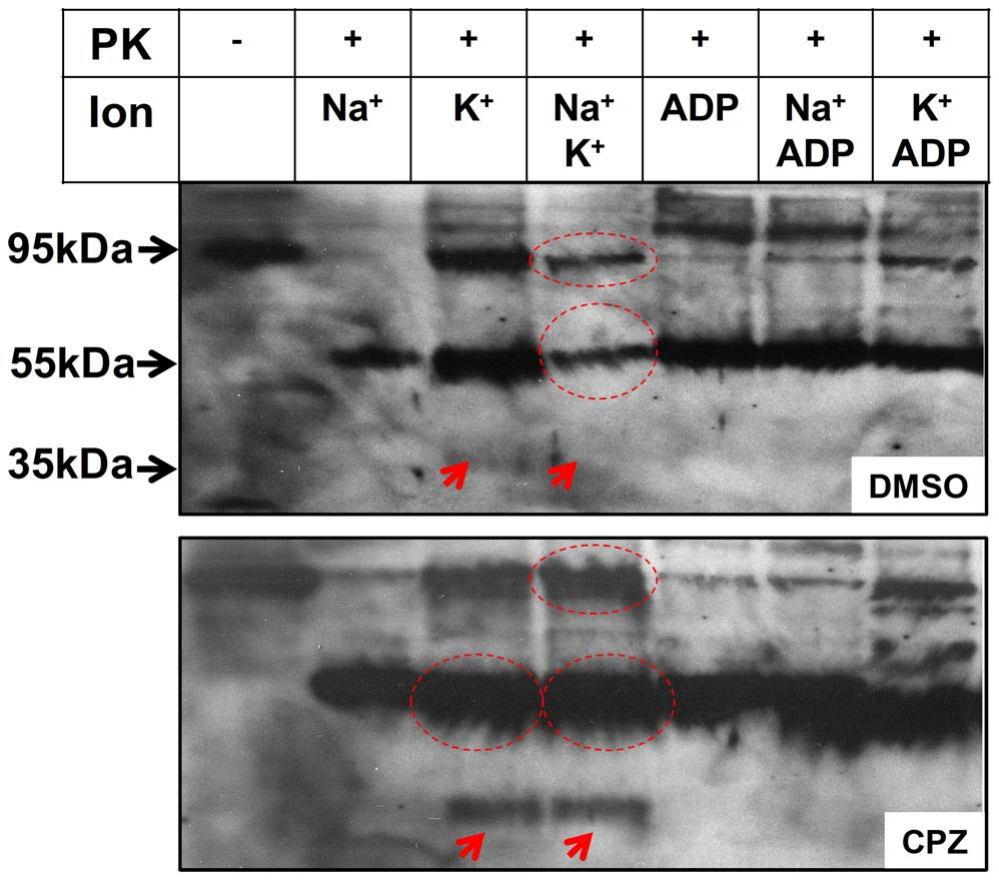
Proteinase K cleavage. Pig kidney membranes were incubated in 25/or 1 mM ADP, as indicated. The samples were treated with PK (PK/protein ratio ∼1∶50) and the mixture incubated for 40 min at 24°C, and terminated with SDS sample buffer acidified with TCA. Proteolytic products were separated using SDS-PAGE and protein fragments on the gel were transferred to PVDF membranes and visualized by Western blotting using NKA1012-1016 antibody. The labels to the left indicate the apparent molecular weights of the fragments, determined by the use of Precision plus protein standards from Bio-Rad, Cat#161-0363.

Cleavage with PK of the cytoplasmic domains of the α-subunit was used as a tool to investigate the effect of CPZ on the conformation of pig kidney Na^+^,K^+^-ATPase. In the presence of occluded ions, a specific cleavage pattern is obtained as proteolytic sites at distinct locations are protected and others are exposed. In the absence of occluded ions, this protection is absent and all of the cytoplasmic domains of the α-subunit are completely cleaved. To this end, membrane-bound enzyme was incubated in the presence of different ion combinations with and without 50 µM CPZ, before the addition of PK (Fig. 6). Fragments were separated by SDS-PAGE and visualized by Western blotting using a specific antibody to the C-terminus of the α-subunit. In control samples, the C-terminal half of the α-subunit (a fragment of 55 kDa starting at an area close to the phosphorylation site in the P domain to the C-terminal end of the α-subunit), containing M5-M10, is accumulated in the presence of K^+^ alone, whereas in the presence of Na^+^ or Na^+^ and K^+^, the level of the 55 kDa fragment was largely reduced (Fig. 6, compare lanes labeled Na^+^, K^+^, and Na^+^ plus K^+^). Parallel experiments where cleavage products were stained with Coomassie blue indicated that the 55 kDa is almost completely cleaved to smaller fragment (data not shown). This indicates that the C-terminal half of enzyme incubated in the presence of Na^+^ alone is less stable compared to the K^+^-bound enzyme, and that the presence of Na^+^ together with K^+^ abrogates the protection provided by K^+^. In samples treated with CPZ and then subjected to PK cleavage, in contrast, the 55 kDa fragment was protected in the presence of Na^+^ alone or in the presence of Na^+^ and K^+^. In addition, CPZ treatment exposed a K^+^-specific new cleavage site that produced a 35 kDa fragment. The 35 kDa fragment is likely a result of cleavage of the 55 kDa fragment at the area connecting the N and P domains [30]. Finally, in the presence of ADP which stabilizes a compact cytoplasmic headpiece structure, the α-subunit was protected in the presence of either Na^+^ or K^+^ and CPZ still had an appreciable protecting effect.

### Trypsin cleavage

We sought to gain more information on the conformation of the control and CPZ modified enzymes by employing trypsin digestion, which produces well-defined cleavages at specific sites in the α-subunit in the presence of either Na^+^ or K^+^ ions [29]. Hence, we determined the cleavage patterns of the pig kidney α-subunit at different pH, and in the presence of 50 mM of Na^+^, K^+^ or guanidinium ions (Gua^+^). Gua^+^ derivatives have earlier been shown to antagonize Na^+^ binding to Na^+^,K^+^-ATPase [34], [35]. Gua^+^ was later employed to investigate cation selectivity of the Na^+^,K^+^-ATPase. From electrical measurements it has been hypothesized that Gua^+^ is accommodated in the third Na^+^ site, as it produces voltage-dependent inhibition of an outward current in the presence of extracellular K^+^ and it permeates through the Na^+^ unique site, producing inward current at negative membrane potentials. These two features characterize Na^+^ interaction with a high field access channel in the pump [36], [37] and hence Gua^+^ is thought of as a Na^+^ congener at the Na^+^ specific site.

**Figure 7:**
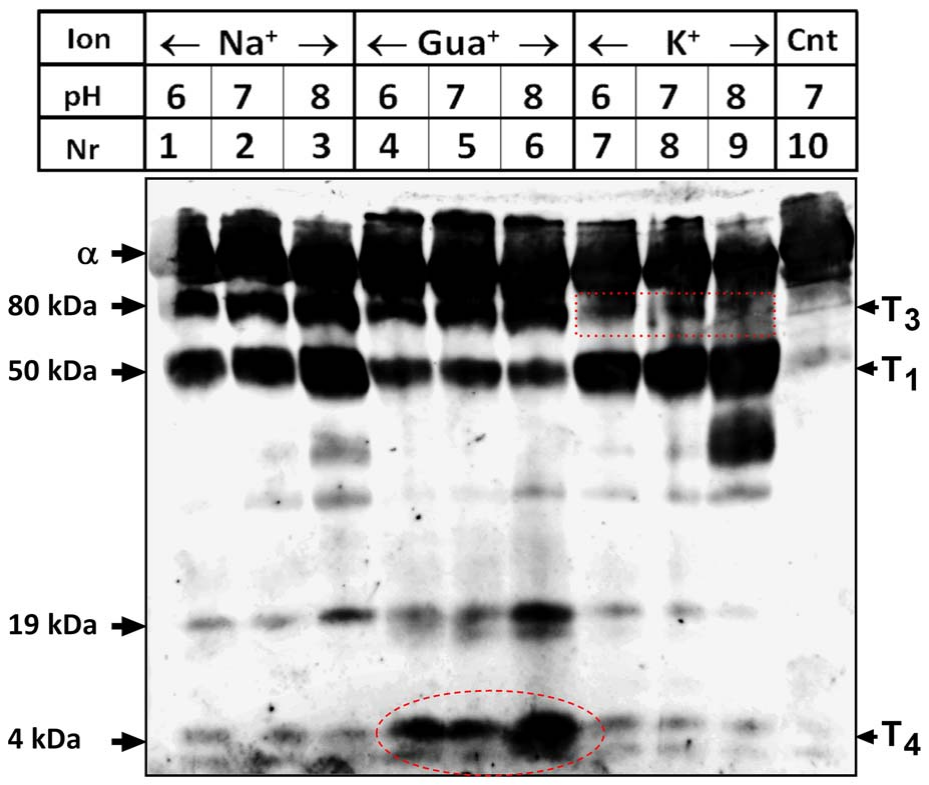
Trypsin cleavage in the presence of substrate ions. Trypsin cleavage of pig kidney Na^+^,K^+^-ATPase was performed in the presence of Tris buffer and 50 mM of NaCl, Gua^+^ hydrochloride, or KCl. The enzyme (100 µg) was allowed to equilibrate with ions for 30 min at 20°C, following by the addition of trypsin (trypsin/protein ratio ∼1∶100). In controls (Cnt), water replaced trypsin. The reaction was allowed to proceed for 40 min and was terminated using SDS sample buffer acidified with TCA. Protein fragments were resolved using SDS-PAGE and visualized by Western blotting using anti KETYY antibody. Each reaction was performed in the presence of Tris buffer with the indicated pH values. Representative of four different experiments is shown. The right labels indicate the symbols of cleavage sites introduced by Jørgensen [29], together with the new T_4_ site. Note that the very faint bands appearing at 4 kDa in the presence of Na^+^ or K^+^ likely represent conformational fluctuations of the protein during incubation with the protease (Ref. [58]).

**Figure 8:**
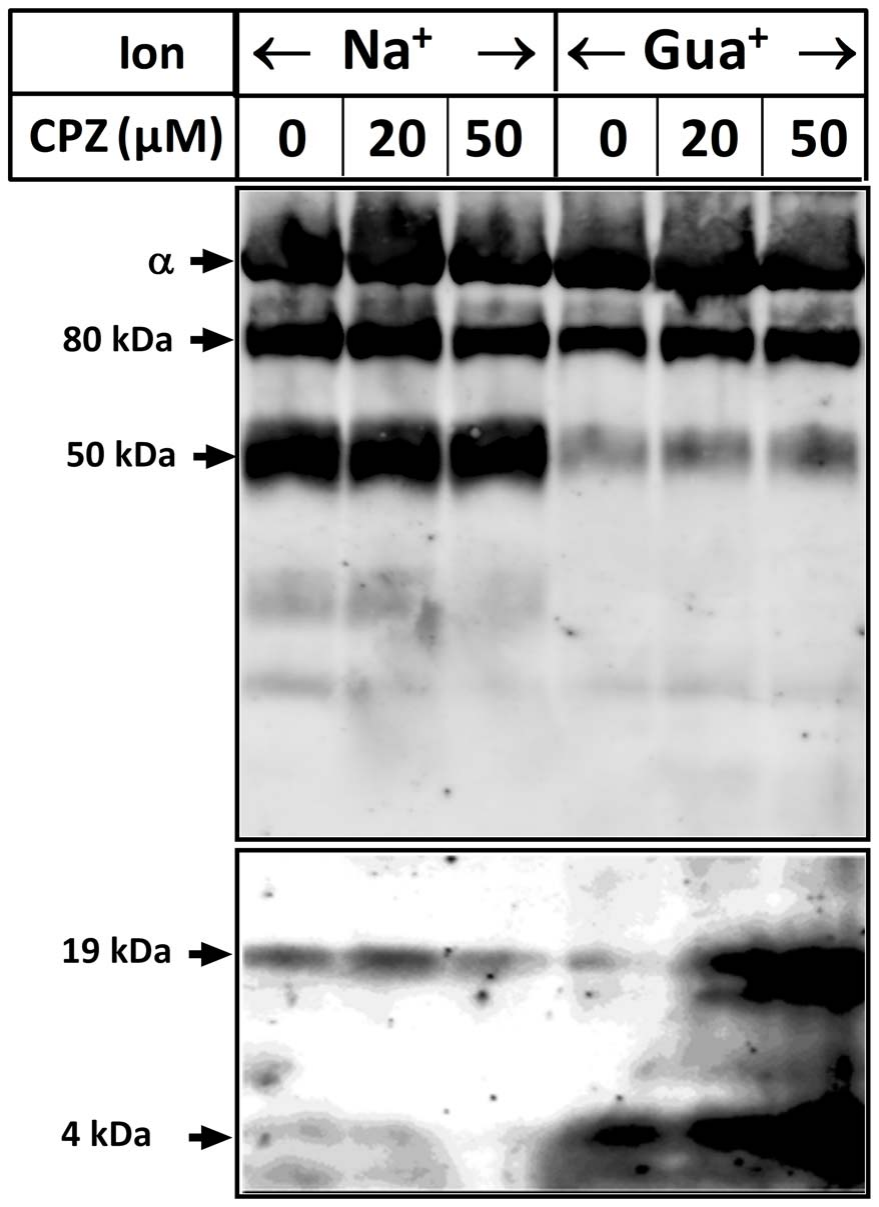
Effect of CPZ on cleavage of the C-terminal tail. Trypsin treatment, SDS-PAGE, and immunoblotting were performed as in Fig. 7. The effect of CPZ on accumulation of the C-terminal fragment was investigated at pH 8 in the presence of either Na^+^ or Gua^+^. Note the significant difference between Na^+^ and Gua^+^ on the accumulation of the 55 kDa fragment, occurring at the middle of the α-subunit (T_1_ site, Ref. [29]). The anomalous migration of the 4 kDa peptide is possibly due to its polar properties and its small molecular mass. Note that, due to the difference in staining intensity, the upper part of the immunoblot was made less intense.

In the presence of Na^+^, cleavage at both T_3_ and T_1_ sites [29] occurred, whereas cleavage occurred almost exclusively at T_1_ in the presence of K^+^ (Fig. 7). Gua^+^ produced cleavage preferentially at T_3_, i.e. a similar cleavage pattern as expected for the E_1_(Na^+^) form [29]. A conformation with exposed T_3_ and substantially protected T_1_ is apparently a novel one stabilized by Gua^+^ binding. Importantly, in the presence of Gua^+^, a short peptide was exposed to cleavage by trypsin especially at pH 8 (site T_4_). This fragment is derived from the C-terminus of the α-subunit, as evidenced from its positive reaction with the KETYY antibody. Edmann degradation of this short peptide after excision from PVDF membranes yielded the sequence “^1006^PGGXVE”, a sequence immediately preceding the KETYY motif, demonstrating unambiguously that the fragment arises from the C-terminus of the α-subunit as a consequence of trypsin cleavage. When the effect of CPZ on trypsin cleavage of the α-subunit at this domain was examined we found that CPZ increased, in a concentration-dependent manner, the exposure of the tryptic site T_4_ at the C-terminus (Fig. 8). The exposure of the C-terminal tail to trypsin cleavage is specific for Gua^+^ and is not observed in the presence of Na^+^ (Fig. 8, and see Discussion).

Another intriguing observation is the appearance of a 19 kDa fragment in the presence of Gua^+^ at pH 8 (Figs. 7 and 8). This fragment is most likely identical to a well-known C-terminal third of the α-subunit starting at N831 (see Discussion) produced by incubation of the membrane-bound enzyme with high trypsin concentrations at 37°C [38].

### Oxonol VI fluorescence measurements

**Figure 9:**
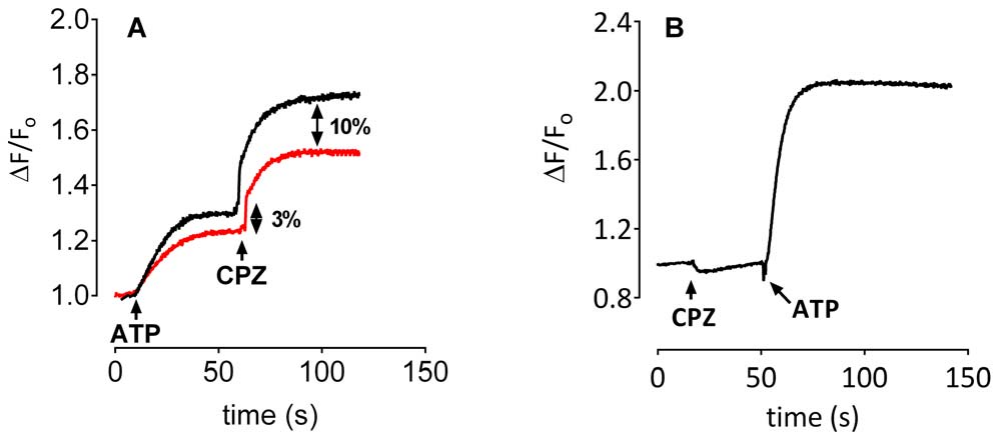
Effect of CPZ on oxonol VI fluorescence under Na^+^,Na^+^ exchange conditions. Oxonol VI fluorescence was measured as described in Materials and Methods. The working volume in the cuvette throughout the measurement was 2–2.1 ml. The cuvette contained 30 mM histidine, 30 mM NaCl, 2 mM MgCl_2_, and 20–30 µl proteoliposomes, and the experiment was started with the addition of 75 µM ATP. After reaching steady state, 20 µM CPZ dissolved in DMSO was added. **A**. Shown is the effect of addition of ATP and CPZ in the absence (black) or in the presence (red) of 25 µM ADP. The numbers indicate the percentage decrease in maximum fluorescence occurred by pretreatment with 25 µM ADP. Representative of four independent experiments is shown. **B**. The fluorescence response was measured as in panel A, but CPZ was added first, followed by the addition of ATP, as indicated by the arrows.

Investigation of changes in the fluorescence of oxonol VI was used to gain information on the effect of CPZ on Na^+^ translocation via the pump. ATP-dependent transport of positive charge from the medium into the liposomal lumen, establishing an inside positive membrane potential, is detected by an increase in oxonol VI fluorescence [39]. As seen from Fig. 9A, addition of ATP in the presence of 30 mM Na^+^ on both sides of the membrane (3Na^+^:2Na^+^ exchange conditions) resulted in an expected increase in fluorescence (Fig 9A, black), indicative of electrogenic Na^+^ exchange with net influx of Na^+^ into the liposomes through reconstituted inside-out Na^+^,K^+^-ATPase molecules (cellular efflux). The fluorescence level reaches a plateau as the potential difference attains a level high enough to impair Na^+^ release from the E_2_P(Na^+^) form. Addition of CPZ at the plateau phase resulted in ∼100% increase in fluorescence to a new plateau. The CPZ effect was pump-mediated since CPZ produced no effect on oxonol VI fluorescence in the absence of the protein (data not shown). Furthermore, addition of CPZ before ATP did not produce any change in fluorescence (Fig. 9B); an increase in fluorescence is only observed following the addition of ATP.

Treatment with ADP is expected to decrease the fluorescence response as it increases the backward reactions of the pump cycle by interacting with E_1_P(3Na^+^). Hence, the difference in fluorescence response in the absence and in the presence of ADP gives information on the approximate level of the ADP sensitive conformations. The presence of 25 µM ADP in the medium decreased the maximum fluorescence by ∼3% and 10% following the addition of ATP and CPZ, respectively (Fig. 9A, red).

**Figure 10:**
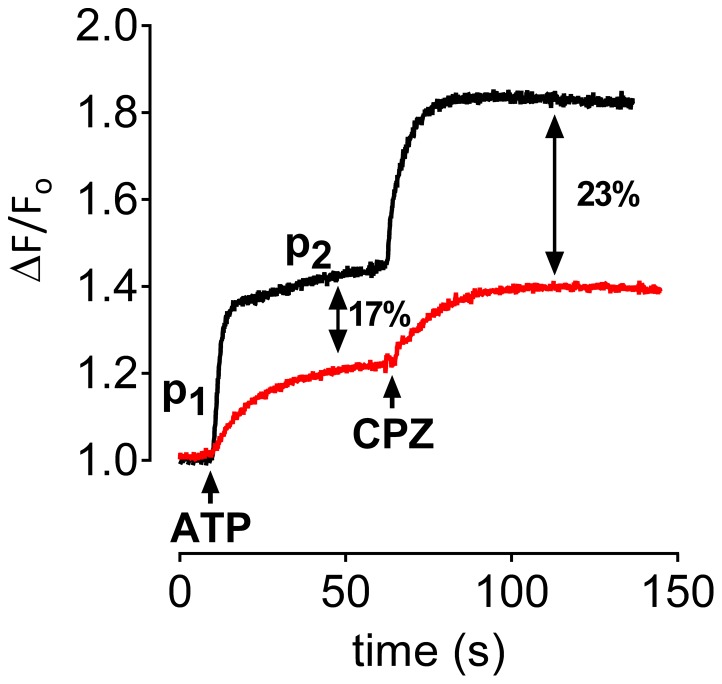
Effect of CPZ on oxonol VI fluorescence under Na^+^,K^+^ exchange conditions. Liposomes containing 30_2_ were used and the experiment was performed as in A. Extravesicular K^+^ was diluted more than 70 folds following addition of liposomes to the medium. The ATP concentration used to start the reaction was 75 µM. The numbers indicate the percentage decrease in maximum fluorescence occurred by pretreatment with 25 µM ADP. P_1_ and P_2_ denote the rapid and the slow phases of fluorescence response occurring in the presence of extracellular (intravesicular) K^+^.

The experiments were also performed with liposomes produced in the presence of 30 mM Na^+^ plus 30 mM K^+^. After dilution in the cuvette, the extracellular side of the inside-out Na^+^,K^+^-ATPase (intravesicular medium) is thus exposed to 30 mM K^+^ whereas the intracellular side (extravesicular medium) is exposed to ∼100–200 µM K^+^. 30 mM Na^+^ was present on both sides of the membrane. Activation of inside-out Na^+^,K^+^-ATPase by addition of 75 µM ATP resulted in an increase in fluorescence (Fig. 10, black). However, the initial rate of fluorescence increase was significantly higher (compare to Fig. 9A) in accordance with activation of a fast 3Na^+^:2K^+^ exchange (phase 1) followed by a much slower phase of fluorescence increase (phase 2, Fig. 10, black). Addition of CPZ at the plateau phase produced an increase in fluorescence as observed in the absence of K^+^ (cf. Fig. 9A, black). This seems at first paradoxical since CPZ inhibits Na^+^,K^+^-ATPase activity. However, after the initial activation of 3Na^+^:2K^+^ exchange by the addition of ATP (Fig. 10, time interval between 10 and 60 seconds on the X-axis), K^+^ is known to be rapidly transported out of the liposomal lumen [39] and the exchange mode is likely shifted to Na^+^ exchange. Therefore, at the time of CPZ addition the increase in fluorescence is likely due to activation of Na^+^ efflux as with the experiments without K^+^ (Fig. 9A). The rapid elimination of intravesicular K^+^ is also indicated by the slow-down from the initial very fast increase in the rate of fluorescence after ATP addition to the much slower subsequent one attained after CPZ addition. Pretreatment with 25 µM ADP resulted in a significant decrease in maximum fluorescence (Fig. 10, red) compared to the decrease observed in the absence of K^+^ (Fig. 9A, red). The maximum fluorescence levels were found to decrease by 17% and 23% following the addition of ATP and CPZ, respectively.

**Figure 11:**
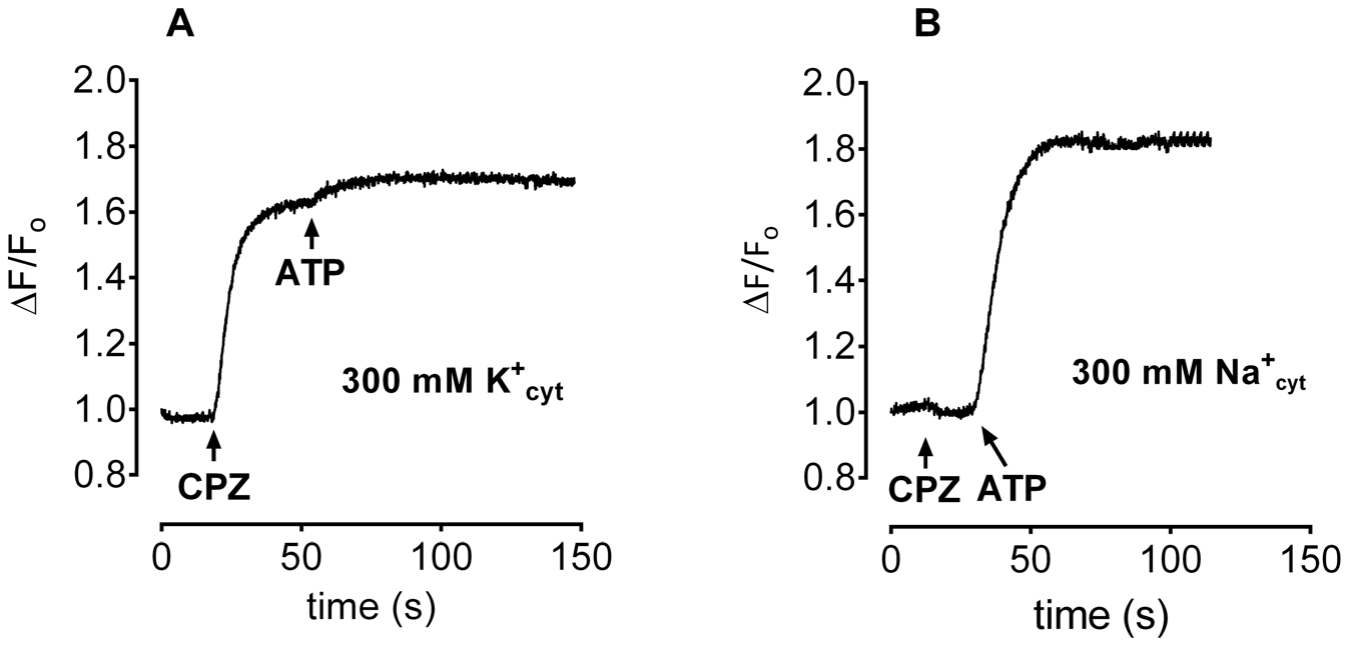
Effect of CPZ on oxonol VI fluorescence in the presence of 10 folds ion gradient across liposomal membrane. Liposomes were equilibrated with either 300(A) or 300 mM NaCl (B). The medium contained 30 mM histidine, 2 mM MgCl_2_ and 20 µl proteoliposomes. CPZ was added first, followed by 75 µl Tris-ATP, as indicated by the arrows.

A surprising effect was observed in experiments where the effect of CPZ was studied in the presence of a high extravesicular concentration (300 mM) of either K^+^ or Na^+^. In the presence of a K^+^ gradient, a substantial increase in fluorescence occurred following addition of CPZ in the absence of ATP (Fig. 11A), and subsequent addition of ATP produced only a very small increase in fluorescence. In contrast, in the presence of 300 mM Na^+^ in the extravesicular medium, CPZ had no effect on fluorescence in the absence of ATP, whereas subsequent addition of ATP increased the fluorescence in a manner similar to that found in Fig. 9A (Fig. 11B). This CPZ effect was not influenced by the addition of digitoxigenin during a pre-incubation period of 5 min (data not shown).

## Discussion

The Na^+^,K^+^-ATPase has been demonstrated in addition to the physiological Na^+^:K^+^ exchange reaction, to catalyze several partial reactions. Thus, in the absence of K^+^, uncoupled Na^+^ efflux, as well as electroneutral and electrogenic Na^+^-Na^+^ exchange have been described (For review see Refs. [40], [41]). We now provide mechanistic information on a modified pump catalyzing a partial reaction-like activity in the presence of K^+^.

### CPZ inhibits active K^+^ transport but allows Na^+^ efflux

Patch clamp experiments (Figs. 1 and 2), ASG II (Fig. 3), and oxonol VI fluorescence measurements (Figs. 9 and 10) confirmed a model in which CPZ impairs Na^+^/K^+^ exchange but allows Na^+^ efflux through the pump. In particular, ASG II data provide strong evidence that the small outward CPZ-induced current is carried by Na^+^ and not by protons. An electro neutral shuttling of Na^+^ on E_1_ can be excluded since the CPZ-modified enzyme carries out electrogenic Na^+^ transport. Under normal conditions, 3Na^+^ leave the cell and 2K^+^ enter. CPZ uncouples this process; K^+^-dependent current is abolished whereas outward Na^+^ current is increased. Hence, in CPZ-modified enzyme active Na^+^ transport seems to be functionally independent from the K^+^ transport although they share two of the three sites. Therefore, the shared sites are either blocked, or their selectivity for K^+^ over Na^+^ is severely increased, resulting in constitutive occupation with K^+^ and inhibition of K^+^-dependent hydrolytic activity. The Na^+^/K^+^ selectivity has been proposed to be the result of highly constrained and tightly juxtaposed Na^+^ binding sites induced by binding of the first Na^+^ ion to the Na^+^ specific site, allowing only small Na^+^ ions to bind [9]. Hence, CPZ may stabilize a similar Na^+^ conformation. Based on the stabilization of K^+^ occluded enzyme [17], CPZ was previously proposed to induce an E_2_ variant. However, the data presented here indicate that CPZ stabilizes an E_1_-like conformation, as indicated from vanadate sensitivity (Fig. 4C) and proteolytic cleavage (Figs. 7 and 8) experiments.

### CPZ modulates ion interaction with the Na^+^,K^+^-ATPase

The presence of K^+^ is essential in protecting the cytoplasmic domains and the 19 kDa fragment of the α-subunit from complete proteolytic cleavage [38], [42], whereas Na^+^ seems to provide little to no protection [43]. Furthermore, occluded K^+^ ions provide more stability and protection against thermal denaturation of the membrane-bound Na^+^,K^+^-ATPase compared to Na^+^ [44], indicating that the K^+^ conformation is more stable than the Na^+^ conformation. PK cleavage experiments show that the complex containing M5-M10 of the α-subunit (55 kDa fragment) is protected from cleavage in the presence of K^+^ (assumed to be bound in the shared sites) but is further cleaved in the presence of Na^+^ (Fig. 6). CPZ confers stability to the Na^+^ bound enzyme, implying that the drug enhances binding to the shared sites of either Na^+^ or K^+^. Indeed, deletion of the two C-terminal tyrosine residues of the α-subunit was reported to reduce the interaction of ions with the shared sites [14]. To explain the PK data, we believe that K^+^ ions bind stably in the shared sites whereas Na^+^ is able to alternate between the shared sites and the Na^+^ specific site. Under this condition, the shared sites may be transiently empty of ions, making the α-subunit more liable to proteolytic attack. This explanation is consistent with the well-established fact that whereas the K^+^ occluded form is formed spontaneously after incubation of the dephosphorylated enzyme with K^+^/Rb^+^ [45], the Na^+^ occluded form is not observed unless the enzyme is phosphorylated from ATP [46].

*p*NPPase activity is stimulated by K^+^ acting from the intracellular side of the membrane [47]. Na^+^ stimulates *p*NPP hydrolysis only in the presence of MgATP, where Na^+^ is considered to bind at the low affinity extracellular sites in the E_2_P form. That Na^+^ alone is incapable of stimulating *p*NPPase activity of the dephosphorylated enzyme implies that Na^+^ cannot stably bind to the shared sites (i.e., it cannot act like K^+^). The stimulation by CPZ of *p*NPPase activity in the presence of Na^+^ alone (Fig. 5A) and in the absence of phosphorylation from ATP is in accord with the above suggestion that CPZ enhances Na^+^ binding to the shared sites in a K^+^ like manner, allowing for net *p*NPP hydrolysis, consistent with our PK data.

Gua^+^ has been suggested to permeate through the Na^+^ specific site [36], [37]. With its large ionic radius (2.2 Å compared to 0.95 Å for Na^+^), it is not possible for Gua^+^ to fit in the space defining the Na^+^ specific site in the E_1_·AlF_4_^-^·ADP·3Na^+^ structure. However, the size, or the exact location of the third Na^+^ site may be slightly different in the E_1_3Na^+^ conformation. Kanai *et al.* found ∼1.4 Å shift in the position of the third Na^+^ in the two protomers in the crystal [9], and they have proposed that inclination of M5 would play a critical role in adjusting the size of the Na^+^ specific site to only accommodate a Na^+^ ion. Hence, changing the position of M5 would allow binding of ions larger than Na^+^. Our tryptic cleavage experiments indicate that we are dealing with a Gua^+^ “occluded” form. Incubation with trypsin in the presence of ions that are not occluded would not produce the specific cleavage pattern seen in Figs. 7 and 8. That Gua^+^ alone produces an exclusive cleavage at T_3_ infers that occlusion at this single site produces the conformational change defining the E_1_ conformation. It is tempting to note that trypsin produces an exclusive cleavage at T_1_ in the presence of K^+^, whereas in the presence of Na^+^ cleavage at both T_3_ and T_1_ is observed.

### Role of the C-terminal tail

As mentioned above, changing the position of M5 would create space for the binding of larger ions to the Na^+^ specific site. Indeed, the C-terminal tail was proposed to critically regulate M5 movements. In fact, M10, from which the C-terminal tail extends, contains a relatively large number of polar amino acids, indicating its loose association with the membrane. Hence, it is noteworthy to mention that temperature, which strongly affects the inhibition of the pump by CPZ (Fig. 4A), has been shown to affect movements of the C-terminal third of the α-subunit, leading to exposure of the extracellular loops between M7M8 and M9M10 [48]. Hyperthermia has even been shown to dissociate all four M7M10 membrane spans from the membrane core to the extracellular medium [49], [50].

**Figure 12:**
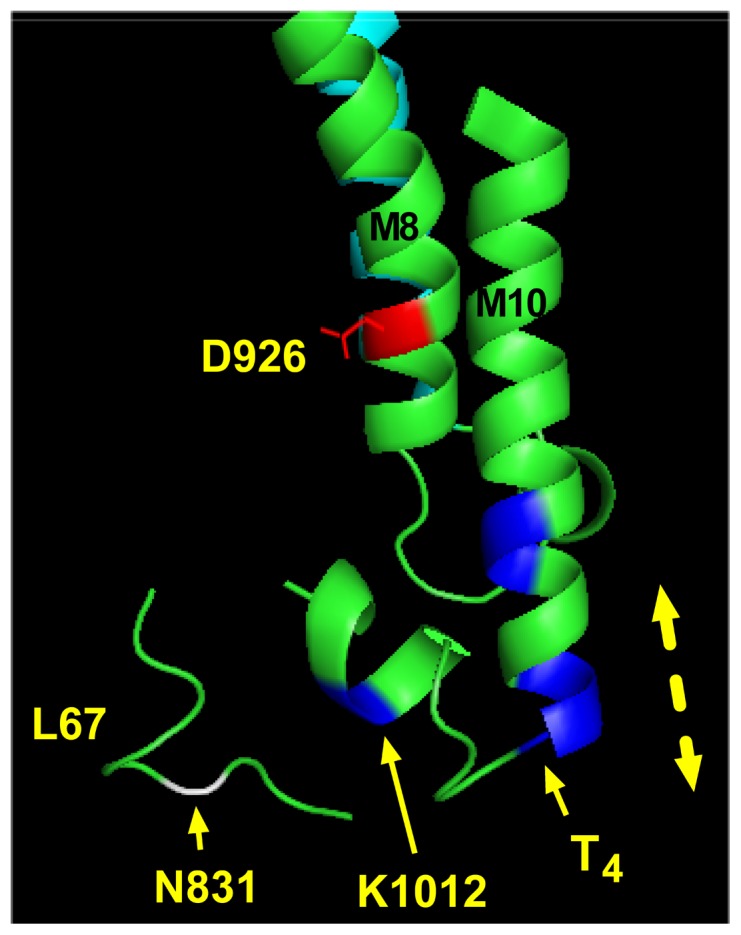
Proteolytic cleavage site in the E_1_ structure of the Na^+^,K^+^-ATPase. Architecture of the cytoplasmic loop between membrane spans 6 and 7, as well as the cytoplasmic part of membrane spans 8–10 together with the C-terminal extension in the E_1_·AlF_4_^−^·ADP·3Na^+^ (PDB accession nr 3WGU). The figure was made using Pymol (www.pymol.com). D926, which coordinates Na^+^ in the unique site, is shown in red. Part of the intracellular loop between membrane spans 6 and 7 (L67) is shown. This part contains an asparagine residue (N831, shown in white), cleavage at which produces the C-terminal 19 kDa fragment of the α-subunit [Ref. 38]. Positive amino acids in the C-terminal part of the α-subunit are shown in blue. T_4_ indicates the cleavage site described in this study, occurring between R1005 and P1006, as evidenced from Edman degradation. Part of M9, shown in cyan, appears behind M8. The dashed double-headed arrow indicates hypothetical movements of M10 that would result in deflection of the C-terminal tail and consequently modification of the ion binding sites.

We have provided direct evidence for a CPZ-mediated conformational change that exposes the C-terminal domain of the α-subunit to trypsin cleavage (as shown in Fig. 8) in the presence of Gua^+^, an ion that was previously shown to permeate the Na^+^ specific site [36], [37]. Fig. 12 shows the location of the T_4_ site in the crystal structure of the pig kidney enzyme in the E_1_·AlF_4_^-^·ADP·3Na^+^ form (PDB accession nr 3WGU, Ref. [9]). The three C-terminal arginine residues, R1003-R1005, build the last C-terminal turn in M10. A similar architecture is also present in the E_2_·MgF_4_^2-^·2K^+^ structure [7]. Proteolytic data (Figs. 7 and 8) suggest that the distal part of M10 is buried in the membrane but is exposed to trypsin attack by a conformational change. The major factor that produces exposure of the C-terminal tail was found to be Gua^+^. The effect of Gua^+^ is enhanced by either high pH (Fig. 7) or CPZ (Fig. 8). Surprisingly, the effects seen in the presence of Gua^+^ were not reproduced by Na^+^. A similar paradox was indeed reported in the study by Ratheal et al. [37]; H^+^ and Gua^+^ were found to leak through site III in the pump whereas Na^+^ did not. This is likely related to the fact that Na^+^ can bind both the shared and the specific site whereas Gua^+^ cannot. If binding to the shared sites controls access to the Na^+^ specific site, the unique effect of Gua^+^ may be reconciled.

Exposure of the C-terminal tail to trypsin cleavage could be explained if it is assumed that M10 moves toward the cytoplasm during conformational changes (see dashed arrow in Fig. 12). In principle, modifying the position of the C-terminal tail would regulate the inclination of M5 and thereby ion selectivity at the shared sites. Distortion of the K^+^ sites may explain the strong modification of K^+^ interaction with the pump. Whether CPZ produces direct effects on the movement of M10 is the subject of future investigations. The presence of membrane-embedded arginine residues in the distal part of the α-subunit's M10 is intriguing, as it may suggest a role of electrostatic interactions in regulating movements of the domain. Biophysical studies will be required to confirm this. Finally, it appears that the loop containing N831, cleavage at which produces the 19 kDa C-terminal fragment, is closer to the T_4_ site in the structure representing the E_1_ form (Fig. 12, Ref. 9) than in the structure representing the E_2_ form [7]. This may explain why we see the 19 kDa fragment after cleavage at 24°C in the presence of Gua^+^.

### Uncoupling as an intrinsic property of P-type pumps

The uncoupling produced by CPZ could be accomplished if we assume that Na^+^ in the unique site exits through a different pathway than that adopted by ions in the shared sites, allowing Na^+^ transport independent of K^+^ transport. Such a model is supported by literature date. Transient pump currents initiated by voltage jumps indicated that two Na^+^ ions are released to the outside by a mechanism distinct from the mechanism that releases the first Na^+^ [51]. Gua^+^ was suggested to permeate through site III under conditions where transport through the shared sites is impaired [37]. In early studies, the coupling ratio of the pump was found to decrease from 1.5 to ∼ 0.5 at low cytoplasmic Na^+^ concentrations (180 µM) and the author concluded that the enzyme can function with less than three sites filled with Na^+^ [18]. Intriguingly, studies on mutants (numbering according to the pig kidney α-subunit) Glu779→Ala [52] and Phe786→Leu [53] revealed information that strongly support our conclusion. Glu779 coordinates one of the shared sites, whereas Phe786 is located close to the extracellular face of the membrane away from the ion binding sites. Both mutations mimic removal of the functional carboxyl group or the aromatic ring, respectively. Interestingly, these mutations produced enzyme sustaining higher Na^+^-ATPase activity compared to the wild-type. Na^+^ was shown to dephosphorylate the enzyme at higher rate (implying that Na^+^ acts in a K^+^-like manner) and K^+^ inhibited ATP hydrolysis owing to unusually stable K^+^ occluded enzyme. Removal of the aromatic ring of Phe786 is not expected to directly affect ion binding sites, hence, altered position of M5 likely accounts for the modified Na^+^/K^+^ selectivity reported earlier [53].

In the presence of a high extravesicular (intracellular) K^+^ concentration, CPZ induces an electrogenic component that is ATP-independent (Fig. 11A) showing that it is not associated with active pumping. A similar response is not observed in the presence of a Na^+^ gradient (Fig. 11B). This difference is quite dramatic and the question is whether this additional component is associated with enzyme mediated ion-specific passive transport across the membrane? It has previously been shown that the Na^+^,K^+^-ATPase provides a K^+^ channel across the membrane in the presence of palytoxin [54]. However, early experiments [17] indicated that CPZ increases K^+^ binding to the membranes, suggesting a modified gating mechanism involving increased rate of K^+^ occlusion, or decreasing rate of K^+^ deocclusion, or both. It is, therefore, inconceivable that CPZ impairs active K^+^ transport by making the enzyme leaky to K^+^. An explanation to the ATP-independent increase in fluorescence induced by CPZ (Figure 11A) could be the internal dipole of the ATPase itself. Membrane proteins sustaining ‘active’ potentials may produce responses ascribed to changes in the internal dipole of the protein, leading to redistribution of the dye molecules and hence change in fluorescence [55]. Since CPZ acts with a proton [56], addition of K^+^ in the vicinity of protonated sites would increase the fluorescence response of the negatively charged dye. Further studies are required to resolve this K^+^ specific response.

In summary, we have confirmed the uncoupling of the Na^+^,K^+^-ATPase that results in reduction of K^+^ transport with preserved or activated Na^+^ transport using patch clamped myocytes and fluorescence of ASG II. The uncoupling is most likely due to modified Na^+^/K^+^ selectivity. In this regard, it is interesting to note that protonation of acidic residues play a significant role in determination of Na^+^/K^+^ selectivity of the shared sites [57]. The switch of CPZ interaction produced by shift from neutral to basic pH may suggest that CPZ itself acts differently in the protonated and deprotonated states and hence the drug exerts different effects at neutral and basic pH (Fig.4B). Although the present results cannot unequivocally discriminate between our different proposals for the CPZ effects, M10 and the tail it carries likely play a pivotal role in the CPZ dependent regulation of K^+^ interaction. Further studies are warranted to investigate the molecular mechanism(s) whereby ion selectivity is regulated; especially those responsible for the active transport of K^+^ into the cell.

## References

[1] Geering K (2008) Functional roles of Na^+^,K^+^-ATPase subunits. Curr Opin Nephrol Hypertens 17: 526–532. 18695395 10.1097/MNH.0b013e3283036cbf

[2] Kaplan JH (2002) Na^+^,K^+^-ATPase. Ann Rev Biochem 71: 511–535. 12045105 10.1146/annurev.biochem.71.102201.141218

[3] Lingrel JB (2010) The physiological significance of the cardiotonic steroid/ouabain-binding site of the Na^+^,K^+^-ATPase. Ann Rev Physiol 72: 395–412. 20148682 10.1146/annurev-physiol-021909-135725 PMC3079441

[4] Cornelius F, Mahmmoud YA (2003) Functional modulation of the sodium pump. The regulatory proteins “Fixit”. News Phyiol Sci 18: 119–124. 10.1152/nips.01434.2003 12750449

[5] Palmgren M, Nissen P (2011) P-type ATPases. Ann Rev Biophys 40: 243–266. 21351879 10.1146/annurev.biophys.093008.131331

[6] Shinoda T, Ogawa H, Cornelius F, Toyoshima C (2009) Crystal structure of the sodium-potassium pump at 2.4 Å resolution. Nature 459: 446–450. 19458722 10.1038/nature07939

[7] Morth JP, Pedersen BP, Toustrup-Jensen MS, Sørensen TL, Petersen J, et al (2007) Crystal structure of the sodium potassium pump. Nature 450: 1043–1049. 18075585 10.1038/nature06419

[8] Nyblom M, Poulsen H, Gourdon P, Reinhard L, Andersson M, et al (2013) Crystal structure of Na^+^,K^+^-ATPase in the Na^+^-bound state. Science 342: 123–127. 24051246 10.1126/science.1243352

[9] Kanai R, Ogawa H, Vilsen B, Cornelius F, Toyoshima C (2013) Crystal structures of a Na^+^ bound Na^+^,K^+^-ATPase preceding the E1P state. Nature 502: 201–206. 24089211 10.1038/nature12578

[10] Holmgren M, Wagg J, Bezanilla F, Rakowski RF, De Weer P, et al (2000) Three distinct and sequential steps in the release of sodium ions by the Na^+^/K^+^-ATPase. Nature 403: 898–901. 10706288 10.1038/35002599

[11] Takeuchi A, Reyes N, Artigas P, Gadsby DC (2008) The ion pathway through the opened Na^+^,K^+^-ATPase pump. Nature 456: 413–416. 18849964 10.1038/nature07350 PMC2585603

[12] Apell HJ, Karlish SJ (2001) Functional properties of Na^+^,K^+^-ATPase, and their structural implications, as detected with biophysical techniques. J memb Biol 180: 1–9. 10.1007/s002320010053 11284199

[13] Toustrup-Jensen MS, Holm R, Einholm AP, Schack VR, Morth JP, et al (2009) The C terminus of Na^+^,K^+^-ATPase controls Na^+^ affinity on both sides of the membrane through Arg935. J Biol Chem 284: 18715–18725. 19416970 10.1074/jbc.M109.015099 PMC2707196

[14] Vedovato N, Gadsby DC (2010) The two C-terminal tyrosines stabilize occluded Na/K pump conformations containing Na^+^ or K^+^ ions. J Gen Physiol 136: 63–82. 20548052 10.1085/jgp.201010407 PMC2894553

[15] Poulsen H, Khandelia H, Morth JP, Bublitz M, Mouritsen OG, et al (2010) Neurological disease mutations compromise a C-terminal ion pathway in the Na^+^/K^+^-ATPase. Nature 467: 99–102. 20720542 10.1038/nature09309

[16] Moran MM, McAlexander MA, Biro T, Szallasi A (2011) Transient receptor potential channels as therapeutic targets. Nat Rev Drug Discov 10: 601–620. 21804597 10.1038/nrd3456

[17] Mahmmoud YA (2008) Capsazepine, a synthetic vanilloid that converts the Na^+^,K^+^-ATPase to Na^+^-ATPase. Proc Natl Acad Sci USA 105: 1757–1761. 18230728 10.1073/pnas.0711838105 PMC2234217

[18] Blostein R (1983) The influence of cytoplasmic sodium concentration on the stoichiometry of the sodium pump. J Biol Chem 258: 12228–12232. 6313645

[19] Einholm AP, Toustrup-Jensen MS, Holm R, Andersen JP, Vilsen B (2010) The rapid-onset dystonia parkinsonism mutation D923N of the Na^+^, K^+^-ATPase alpha3 isoform disrupts Na^+^ interaction at the third Na^+^ site. J Biol Chem 285: 26245–25254. 20576601 10.1074/jbc.M110.123976 PMC2924038

[20] Cornelius F, Skou JC (1985) Na^+^, Na^+^ exchange mediated by Na^+^,K^+^-ATPase reconstituted into liposomes. Evaluation of pump stoichiometry and responses to ATP and ADP. Biochim Biophys Acta 818: 211–221. 2992589 10.1016/0005-2736(85)90572-3

[21] Pavlovic D, McLatchie LM, Shattock MJ (2010) The rate of loss of t-tubules in cultured adult ventricular myocytes is species dependent. Exp Physiol 95: 518–527. 20061354 10.1113/expphysiol.2009.052126

[22] Pavlovic D, Fuller W, Shattock MJ (2007) The intracellular region of FXYD1 is sufficient to regulate cardiac Na^+^,K^+^-ATPase. FASEB J 21: 1539–1546. 17283221 10.1096/fj.06-7269com

[23] Fuller W, Howie J, McLatchie LM, Weber RJ, Hastie CJ, et al (2009) FXYD1 phosphorylation in vitro and in adult rat cardiac myocytes: Threonine 69 is a novel substrate for protein kinase C. Am J Physiol Cell Physiol 296: C1346–C1355. 19339511 10.1152/ajpcell.00523.2008 PMC2692419

[24] Pavlovic D, Hall AR, Kennington EJ, Aughton K, Boguslavskyi A, et al (2013) Nitric oxide regulates cardiac intracellular Na^+^ and Ca^2+^ by modulating Na/K ATPase via PKCε and phospholemman-dependent mechanism. J Mol Cell Cardiol 61: 164–171. 23612119 10.1016/j.yjmcc.2013.04.013 PMC3981027

[25] Despa S, Vecer J, Steels P, Ameloot M (2000) Flourescence lifetime microscopy of the Na^+^ indicator Na^+^ green in HeLa cells. Anal Biochem 281: 159–175. 10870831 10.1006/abio.2000.4560

[26] Klodos I, Esmann M, Post RL (2002) Large-scale preparation of sodium-potassium ATPase from kidney outer medulla. Kid Int 62: 2097–2100. 10.1046/j.1523-1755.2002.00654.x 12427133

[27] Baginsky ES, Foa PP, Zak B (1967) Micro determination of inorganic phosphate, phospholipids, and total phosphate in biological materials. Clin Chim Acta 13: 326–332. 6036717

[28] Mahmmoud YA, Cramb G, Maunsbach AB, Cutler CP, Meischke L, et al (2003) Regulation of Na^+^,K^+^-ATPase by PLMS, the phospholemman-like protein from shark: molecular cloning, sequence, expression, cellular distribution, and functional effects of PLMS. J Biol Chem 278: 37427–37438. 12874284 10.1074/jbc.M305126200

[29] Jørgensen PL, Collins JH (1986) Tryptic and chemotryptic cleavage sites in sequence of α-subunit of Na^+^,K^+^-ATPase from outer medulla of mammalian kidney. Biochim Biophys Acta 860: 570–576. 3017424 10.1016/0005-2736(86)90555-9

[30] Cornelius F, Mahmmoud YA, Toyoshima C (2011) Metal fluoride complexes of Na^+^,K^+^-ATPase: Characterization of fluoride-stabilized phosphoenzyme analogues and their interaction with cardiotonic steroids. J Biol Chem 286: 29882–29892. 21708939 10.1074/jbc.M111.259663 PMC3191029

[31] Cornelius F, Skou JC (1984) Reconstitution of Na^+^, K^+^-ATPase into phospholipid vesicles with full recovery of its specific activity. Biophys Biochim Acta 772: 357–373. 10.1016/0005-2736(84)90153-6 6326830

[32] Mahmmoud YA, Christensen SB (2011) Oleic and linoleic acids are active principles in Nigella sativa and stabilize an E_2_P conformation of the Na^+^,K^+^-ATPase. Fatty acids differentially regulate cardiac glycoside interaction with the pump. Biochim Biophys Acta 1808: 2413–2420. 21767529 10.1016/j.bbamem.2011.06.025

[33] Lamy CM, Chatton J-Y (2011) Optical probing of sodium dynamics in neurons and astrocytes NeuroImage. 58: 572–578. 10.1016/j.neuroimage.2011.06.074 21763440

[34] David P, Mayan H, Cohen H, Tal DM, Karlish SJD (1992) Guanidinium derivatives act as high affinity antagonists of Na^+^ ions in occlusion sites of Na^+^,K^+^-ATPase. J Biol Chem 267: 1141–1149. 1309763

[35] Or E, David P, Shainskaya A, Tal DM, Karlish SJD (1993) Effect of competitive sodium-like antagonists on Na^+^,K^+^-ATPase suggest that cation occlusion from the cytoplasmic surface occurs in two steps. J Biol Chem 268: 16929–16937. 8394324

[36] Yaragatupalli S, Olivera JF, Gatto C, Artigas P (2009) Altered Na^+^ transport after an intracellular α-subunit deletion reveal strict external sequential release of Na^+^ from the Na^+^/K^+^ pump. Proc Natl Acad Sci USA 106: 15507–15512. 19706387 10.1073/pnas.0903752106 PMC2741281

[37] Ratheal IM, Virgin GK, Yu H, Roux B, Gatto C, et al (2010) Selectivity of externally facing ion-binding sites in the Na/K pump to alkali metals and organic cations. Proc Natl Acad Sci USA 107: 18718–1872. 20937860 10.1073/pnas.1004214107 PMC2972997

[38] Karlish S, Goldshleger R, Stein WD (1990) A 19-kDa C-terminal tryptic fragment of the alpha chain of Na^+^/K^+^-ATPase is essential for occlusion and transport of cations. Proc Natl Acad Sci USA 87: 4566–4570. 2162048 10.1073/pnas.87.12.4566 PMC54157

[39] Apell HJ, Bersch B (1987) Oxonol VI as an optical indicator for membrane potentials in lipid vesicles. Biochim Biophys Acta 903: 480–494. 2444259 10.1016/0005-2736(87)90055-1

[40] Kaplan JH (1983) Sodium ions and the sodium pump: transport and enzymatic activity. Am J Physiol Gastrointes Liver Physiol 245: G327–G333. 10.1152/ajpgi.1983.245.3.G327 6311029

[41] Cornelius F (1991) Functional reconstitution of the sodium pump. Kinetics of exchange reactions performed by reconstituted Na^+^,K^+^-ATPase. Biochim Biophys Acta 1071: 19–66. 1848452 10.1016/0304-4157(91)90011-k

[42] Shainskaya A, Karlish SJD (1994) Evidence that the cation occlusion domain of Na/K-ATPase consists of a complex of membrane-spanning segments. Analysis of limit membrane-embedded tryptic fragments. J Biol Chem 269: 10780–10789. 8144667

[43] Capasso JM, Hoving S, Tal DM, Goldshleger R, Karlish SJ (1992) Extensive digestion of Na^+^,K^+^-ATPase by specific and nonspecific proteases with preservation of cation occlusion sites. J Biol Chem 267: 1150–1158. 1309764

[44] Kaufman SB, Luis González-Flecha F, González-Lebrero RM (2012) Opposing effects of Na^+^ and K^+^ on the thermal stability of Na^+^,K^+^-ATPase. J Phys Chem B 116: 3421–3429. 22283598 10.1021/jp2124108

[45] González-Lebrero RM, Kaufman SB, Montes MR, Nørby JG, Garrahan PJ, et al (2002) The Occlusion of Rb^+^ in the Na^+^/K^+^-ATPase. I. The identity of occluded states formed by the physiological or the direct routes: occlusion/deocclusion kinetics through the direct route. J Biol Chem 277: 5910–921. 11739377 10.1074/jbc.M105886200

[46] Glynn IM, Hara Y, Richards ED (1984) The occlusion of sodium ions within the mammalian sodium-potassium pump: its role in sodium transport. J Physiol 351: 531–554. 6086905 10.1113/jphysiol.1984.sp015261 PMC1193133

[47] Drapeau P, Blostein R (1980) Interaction of K^+^ with Na^+^,K^+^-ATPase. Orientation of K^+^-phosphatase sites studied with inside-out red cell membrane vesicles. J Biol Chem 255: 7827–7834. 6249815

[48] Donnet C, Arystarkhova E, Sweadner KJ (2001) Thermal denaturation of the Na^+^,K^+^-ATPase provides evidence for α-α oligomeric interaction and γ subunit association with the C-terminal domain. J Biol Chem 276: 7357–7365. 11099502 10.1074/jbc.M009131200

[49] Goldshleger R, Tal DM, Karlish SJD (1995) Topology of the alpha-subunit of Na^+^,K^+^-ATPase based on proteolysis. Lability of the topological organization. Biochemistry 34: 8668–8679. 7612607 10.1021/bi00027a016

[50] Arystarkhova E, Gibbons DL, Sweadner KJ (1995) Topology of the Na^+^,K^+^-ATPase. Evidence for externalization of a labile transmembrane structure during heating. J Biol Chem 270: 8785–8796. 7721785 10.1074/jbc.270.15.8785

[51] Ding Y, Hao J, Rakowski RF (2011) Effects of oligomycin on transient current carried by Na^+^ translocation of Bufo Na^+^,K^+^-ATPase expressed in Xenopus Oocytes. J Memb Biol 243: 35–46. 10.1007/s00232-011-9390-6 21877177

[52] Vilsen B (1995) Mutant Glu781→Ala of the rat kidney Na^+^,K^+^-ATPase displays low cation affinity and catalyzes ATP hydrolysis at a high rate in the absence of potassium ions. Biochemistry 34: 1455–1463. 7827094 10.1021/bi00004a041

[53] Vilsen B (1999) Mutant Phe788→Leu of the Na^+^,K^+^-ATPase is inhibited by micromolar concentrations of potassium and exhibits high Na^+^-ATPase activity at low sodium concentrations. Biochemistry 38: 11389–11400. 10471289 10.1021/bi990951t

[54] Rossini GP, Bigiani A (2011) Palytoxin action on the Na^+^,K^+^-ATPase and the disruption of ion equilibria in biological systems. Toxicon 57: 429–439. 20932855 10.1016/j.toxicon.2010.09.011

[55] Cooper CE, Bruce D, Nicholls P (1990) Use of Oxonol V as a probe of membrane potential in liposomes containing cytochrome oxidase in the sub mitochondrial orientation. Biochemistry 29: 3859–3865. 2162199 10.1021/bi00468a009

[56] Yamamura H, Ugawa S, Ueda T, Nagao M, Shimada S (2004) Capsazepine Is a Novel Activator of the δ Subunit of the Human Epithelial Na^+^ Channel. J Biol. Chem 279: 44483–44489. 15308635 10.1074/jbc.M408929200

[57] Yu H, Ratheal IM, Artigas P, Roux B (2011) Protonation of key acidic residues is critical for the K^+^ selectivity of the NaK pump. Nature Struc Mol Biol 18: 1159–1163. 10.1038/nsmb.2113 PMC3190665 21909093

[58] Inesi G, Lewis D, Toyoshima C, Hirata A, de Meis L (2008) Conformational fluctuations of the Ca^2+^-ATPase in the native membrane environment. Effects of pH, temperature, catalytic substrates, and thapsigargin. J Biol Chem 283: 1189–1196. 17993458 10.1074/jbc.M707189200

